# Autoinflammation leading to autoimmunity in adult-onset Still’s disease: more than simple coincidence?

**DOI:** 10.1186/s40001-021-00581-z

**Published:** 2021-09-20

**Authors:** Larissa Valor-Méndez, Bernhard Manger, Alexander Cavallaro, Stephan Achenbach, Georg Schett, Jürgen Rech

**Affiliations:** 1 Department of Internal Medicine 3, Rheumatology and Immunology, Friedrich Alexander University Erlangen-Nuremberg and Universitätsklinikum Erlangen, Ulmenweg 18, Erlangen, Germany; 2 Deutsches Zentrum Für Immuntherapie (DZI), FAU Erlangen-Nürnberg and Universitätsklinikum Erlangen, Erlangen, Germany; 3 Department of Radiology, Friedrich Alexander University Erlangen-Nuremberg and Universitätsklinikum Erlangen, Erlangen, Germany; 4 Department of Internal Medicine 2, Cardiology. Friedrich Alexander University Erlangen-Nuremberg and Universitätsklinikum Erlangen, Erlangen, Germany

**Keywords:** Adult-onset Still’s disease, IL-1 receptor inhibitor, Anakinra, Libman–Sacks endocarditis, Dilated cardiomyopathy, ANTI-beta-1-adrenergic receptor antibodies, Gout

## Abstract

**Background:**

Adult-onset Still’s disease (AOSD) should be considered in the differential diagnosis of patients with endocarditis, with or without a cardiac decompensation.

**Case presentation:**

We report the case of a 68-year-old Caucasian male diagnosed with AOSD after an initial acute manifestation of endocarditis with severe aortic acute manifestation of endocarditis with severe aortic insufficiency. The histological findings revealed Libman–Sacks endocarditis. He was treated with the IL-1 receptor inhibitor anakinra. Two years later the patient developed a symptomatic dilated cardiomyopathy with reduced ejection fraction (23.5%) and functional anti-beta-1-adrenergic receptor antibodies, which was initially treated with plasmapheresis; anakinra was maintained. While his AOSD symptoms responded well, our patient presented with recurrent arthritis in multiple joints, dual-energy CT showed urate deposition compatible with a gouty arthropathy. Over 7 years, he presented with recurrent episodes of arthritis and the adjustment of dosages of colchicine and febuxostat was needed. In 2018, our patient died due to a deterioration of his underlying cardiac disease.

**Conclusions:**

Only two cases with initial endocarditis prior to AOSD diagnosis have been published, and we are not aware of any other cases reporting -β1AR-Ab development with DCM and gout in the setting of AOSD treated with anakinra.

## Background

Adult-onset Still’s disease (AOSD) is an uncommon systemic inflammatory disease on the clinical spectrum of autoinflammatory disorders and is characterized by high fever, arthralgia or arthritis, an evanescent salmon-colored skin rash and leukocytosis with neutrophilia [1]. The concept of a biphasic disease with initially activation of the innate immune system, triggered by danger signals, which then can led to activation of the adaptive immune system if not stopped early effectively is actually the understanding of the pathophysiology [2]. AOSD is a rare disorder with potential cardiac involvement, serosal involvement occurs in 25%-60% of patients and might be severe, while pericarditis, myocarditis, cardiac tamponade are less prevalent. Endocardial involvement is rare, and can present as non-infective endocarditis [1, 3, 4]. The IL-1 receptor antagonist (IL-1Rα) anakinra, which inhibits both IL-1α and IL-1β activity has been approved for the treatment of AOSD [2, 5, 6].

## Case presentation

We report the case of a 68-year-old Caucasian male. On presentation in 2009, he had dyspnea that worsened within 4 weeks, a transesophageal echocardiography demonstrated endocarditis with severe aortic insufficiency. The patient was referred to the cardiology department with ineffective antimicrobial treatment still suffering from intermittent fever, chills, lymphadenopathy and retrospectively myalgia and arthralgia in knees, ankles and shoulders at least for the last 6 months. Pharyngitis and skin rash were denied. The laboratory findings included elevated high C­reactive protein (CRP, 335.2 mg/l), leukocytosis (18.96 × 103/µl with neutrophilia (84%), anemia (8.6 g/dl), thrombocytosis (470 × 103/µl) and elevated serum ferritin (2923 ng/ml). Possible infectious diseases/toxic causes and malignancies were ruled out. He fulfilled the Yamaguchi diagnosis criteria for AOSD [7] at that time point (3 major: leukocytosis > 10,000/mm3 with > 80% polymorphonuclear cells, arthritis lasting over 2 weeks, fever and 2 minor criteria: negative antinuclear antibody/negative rheumatoid factor and lymphadenopathy). Toxic causes, malignancies, infectious diseases (including borreliosis and brucellosis), rheumatic diseases such as vasculitis or systemic erythematous lupus, in the frame of a similar workout as described by Ruscitti et al. were ruled out [8].

**Fig. 1:**
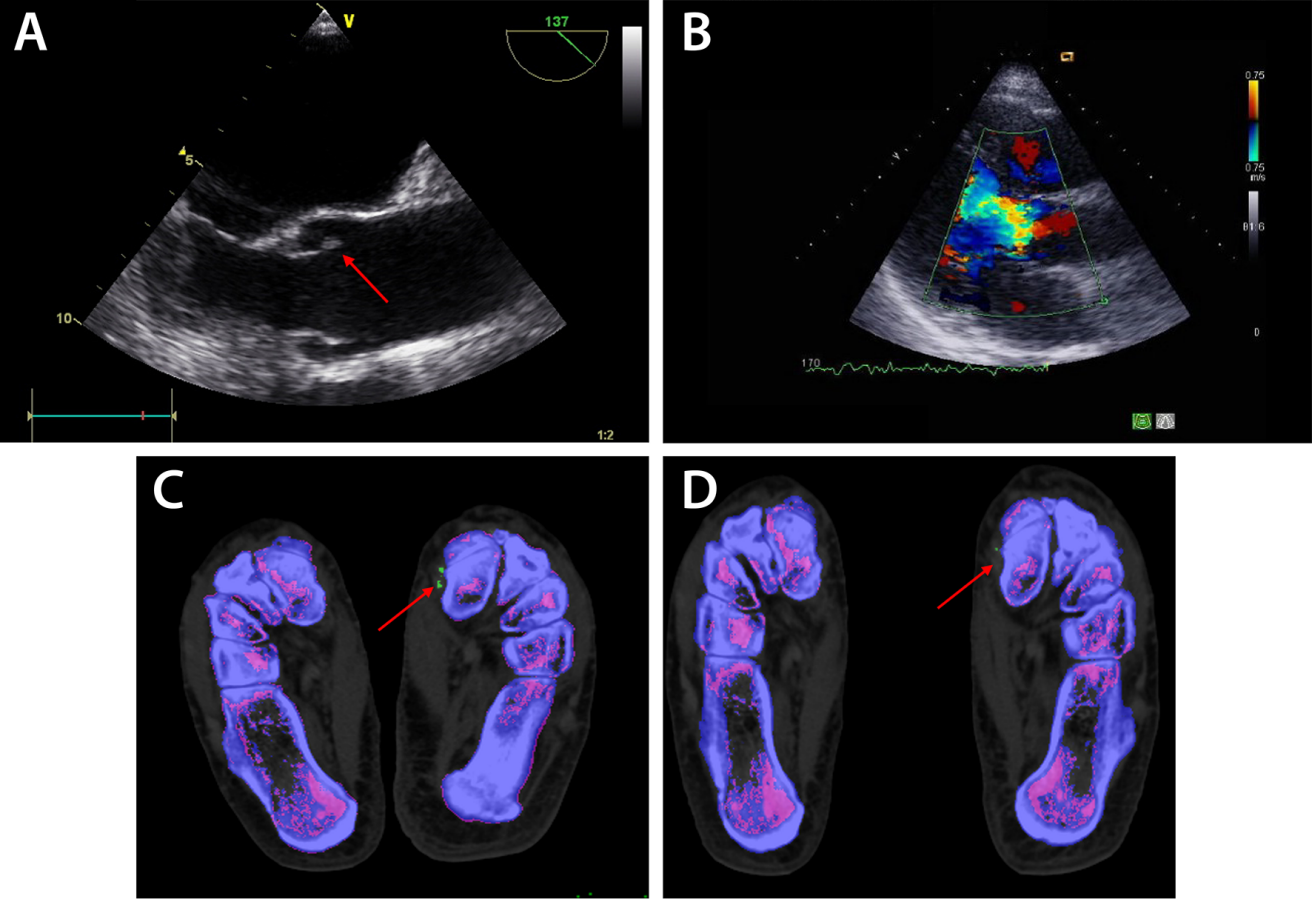
**A**, **B** Two-dimensional transesophageal echocardiography, suspected endocarditis on aortic valve (red arrow) with severe regurgitation seen on color flow imaging (**B**). **C** Urate deposition (green dots marked by red arrow) in dual-energy CT. **D** Five months later, urate depositions (red arrow) were partially reduced

Treatment with the IL-1 receptor inhibitor anakinra (100 mg s.c./d) was started. Immediately a CRP, leukocytosis and ferritin normalization occurred. Preoperatively anakinra was stopped and our patient underwent heart surgery with insertion of a mechanical aortic valve. The microbiological and macroscopic findings demonstrated sterile pocket valve parts, partly translucent with focal polypose-nodular yellowish deposits. The histological findings showed an endocardial duplication with moderate fibrosclerosis and florid inflammation with neutrophilic granulocytes in addition to a lympho-plasmocytic inflammatory infiltrate and isolated eosinophilic granulocytes, fibrin and fibrinous infiltrates; which were highly suspected for Libman–Sacks endocarditis (Fig. 1A, B). Spironolactone und coumarin were added to his routine medication. He had no family history of rheumatic or inflammatory diseases; his treatment included losartan, metoprolol, ramipril und torasemide due to his arterial hypertension and a mild renal insufficiency, he was a former smoker and had a previous knee surgery without complications.

Inflammation parameters increased immediately the day after cardiac surgery, so anakinra was re-started. Two years later the patient developed a symptomatic dilated cardiomyopathy (DCM) with a strongly reduced ejection fraction (23.5%) and functional anti-beta-1-adrenergic receptor antibodies (anti-β1AR-Ab) were found, his AOSD was by that time well controlled and other causes for DCM including cardiac amyloidosis were explored and ruled out. These antibodies might bind to and constitutively stimulate the β1AR to induce β1AR desensitization/downregulation and pathological cardiac remodeling, have been associated with the pathogenesis of DCM and correlate with a poor prognosis [5].

Therefore, we performed nine cycles of daily plasmapheresis, while anakinra was maintained. Additionally, we added two doses of rituximab 1000 mg i.v. each. Both were well tolerated and efficient; our patient recovered to an ejection fraction to 40% and was stable under tight cardiologic monitoring for the next couple of years. After discharge, we initiated treatment with azathioprine 50 mg/day and continued the anakinra therapy.

While his AOSD symptoms were responding well to anakinra, our patient presented another 4 years later with recurrent arthritis of both ankles, related to elevated uric levels acid at 16.5 mg/dl and CRP at 55.6 ml/l. Dual-energy CT (Fig. 1C) showed urate deposition metatarsophalangeal, in the left dorsal tibiotarsal joints, in the tendon attachment of the left tibialis anterior muscle and plantar, marginal sclerosed osteolysis and erosions in the tarsus. Tophaceous gout was diagnosed and treatment with colchicine and febuxostat was started. Five months later, urate depositions were partially reduced, in part constant but was still in the context of the gouty arthropathy (Fig. 1D).

Over 7 years, he presented with recurrent episodes of arthritis and the adjustment of dosages of colchicine and febuxostat was needed. In 2018, our patient died due to a deterioration of his underlying cardiac disease.

## Discussion and conclusions

AOSD and gout are considered polygenetic auto-inflammatory diseases and their clinical manifestations are thought to result from exaggerated activation of innate immune pathways. Both diseases display a recurrent episodic course and respond well to anti-IL-1 treatment. Autoinflammatory diseases may develop chronic disease manifestations that suggest adaptive immune activation as well. In this context, it has been also hypothesized that the development of pathogenic autoantibodies such as anti-β1AR-Ab might occur through autoimmunization. This is consistent with alterations in humoral/cellular immunity, antigen mimicry and with injury-induced release of autoantigens.

At presentation, our patient developed a Libman–Sacks endocarditis. Even under control of the IL-1 pathway with anakinra, he developed anti-β1AR-Ab and a later gouty arthropathy. Was the standard 100 mg/day dose of anakinra not sufficient to suppress IL-1 bioactivity in this specific patient? Might all these episodes be immunologically related? On the one hand, a recent publication describes anti-IL-1 blockade in monogenetic and complex autoinflammatory diseases developing complications and/or a chronic disease course in which different disease mechanisms, including adaptive immune pathways seem to play a role [9]. On the other hand, T-lymphocyte activation via β1-AR autoantibodies causes IL-6 release, which could sustain a vicious cycle. Although the mechanism of initiation of this antibody formation is unknown, activated toll-like receptor-9 might explain the connection between innate and adaptive immunity [9]. We report this case to share our experience, which provides further evidence that AOSD should be considered in the differential diagnosis of patients with endocarditis, with or without a cardiac decompensation. To the best of our knowledge, only two cases with initial endocarditis preceding AOSD diagnosis have been published [3, 10] and we are not aware of other cases reported of anti-β1AR-Ab development with DCM and gout in the frame of AOSD treated with anakinra.

## Abbreviations

AOSD: Adult-onset Still’s disease
IL-1Rα: IL-1 receptor antagonist
CRP: C­reactive protein
DCM: Dilated cardiomyopathy
Anti-β1AR-Ab: Anti-beta-1-adrenergic receptor antibodies
